# Metabolic Associated Fatty Liver Disease Is Associated With an Increased Risk of Severe COVID-19: A Systematic Review With Meta-Analysis

**DOI:** 10.3389/fmed.2021.626425

**Published:** 2021-03-12

**Authors:** Péter Jenő Hegyi, Szilárd Váncsa, Klementina Ocskay, Fanni Dembrovszky, Szabolcs Kiss, Nelli Farkas, Bálint Erőss, Zsolt Szakács, Péter Hegyi, Gabriella Pár

**Affiliations:** ^1^Institute for Translational Medicine, Medical School, University of Pécs, Pécs, Hungary; ^2^Szentágothai Research Center, University of Pécs, Pécs, Hungary; ^3^Doctoral School of Clinical Medicine, University of Szeged, Szeged, Hungary; ^4^Institute of Bioanalysis, Medical School, University of Pécs, Pécs, Hungary; ^5^Division of Gastroenterology, First Department of Medicine, Medical School, University of Pécs, Pécs, Hungary

**Keywords:** SARS-CoV-2, COVID-19, pandemic, prognosis, non-alcoholic fatty liver disease, metabolic associated fatty liver disease

## Abstract

**Background:** The most common pre-existing liver disease, the metabolic dysfunction-associated fatty liver disease (MAFLD) formerly named as non-alcoholic fatty liver disease (NAFLD), may have a negative impact on the severity of COVID-19. This meta-analysis aimed to evaluate if MAFLD or NAFLD are associated with a more severe disease course of COVID-19.

**Methods:** A systematic search was performed in five databases for studies comparing severity, the rate of intensive care unit (ICU) admission, and mortality of COVID-19 patients with and without MAFLD or NAFLD. In meta-analysis, pooled odds ratios (ORs) with 95% confidence intervals (CIs) were calculated.

**Results:** Altogether, we included nine studies in our quantitative and qualitative synthesis. MAFLD was associated with an increased risk of severe COVID-19 compared to the non-MAFLD group (28 vs. 13%, respectively; OR = 2.61, CI: 1.75–3.91). Similarly, in the NAFLD vs. non-NAFLD comparison, NAFLD proved to be a risk factor as well (36 vs. 12%, respectively; OR = 5.22, CI: 1.94–14.03). On the other hand, NAFLD was not associated with an increased risk of ICU admission (24 vs. 7%, respectively; OR = 2.29, CI: 0.79–6.63). We were unable to perform meta-analysis to investigate the association of MAFLD with the rate of ICU admission and with mortality.

**Conclusion:** In conclusion, patients with MAFLD and NAFLD showed a more severe clinical picture in COVID-19. Our results support the importance of close monitoring of COVID-19 patients with MAFLD. Further research is needed to explore the cause of increased severity of COVID-19 in MAFLD.

## Introduction

Severe acute respiratory syndrome coronavirus 2 (SARS-CoV-2) represents a global health challenge. Coronavirus disease 2019 (COVID-19) is mostly a self-limiting disease; however, in some cases, mortality can reach 3–7% (1). The high mortality has been mainly linked to the excessive production of pro-inflammatory cytokines that lead to organ failure, most importantly, acute respiratory distress syndrome (ARDS) (2).

Advanced age and comorbidities, such as hypertension, chronic obstructive pulmonary disease, or cardiovascular diseases are proved risk factors in COVID-19 (1, 3). Patients with elements of metabolic syndrome (MS), such as diabetes, obesity, or hyperlipidemia are more susceptible to infection and also have worse outcomes in COVID-19 (4, 5). MS was found to be associated with chronic low-grade inflammation that compromises the immune system and causes microvascular endothelial dysfunction, which may contribute to poor outcomes in COVID-19 (6, 7).

Metabolic-associated fatty liver disease (MAFLD) and non-alcoholic fatty liver disease (NAFLD) are the most common chronic liver diseases (CLD), which affect about a quarter of the world's adult population (8). Pre-existing liver diseases such as NAFLD or the recently defined MAFLD, as the hepatic manifestations of MS (8), might also be significant risk factors of hospitalization and severity in COVID-19 (9, 10). The MAFLD criteria are based on evidence of hepatic steatosis in addition to one of the following three criteria: overweight/obesity, presence of type 2 diabetes mellitus, and proof of metabolic dysregulation (8).

According to recent publications, the presence of MAFLD and NAFLD may exacerbate the virus-induced inflammatory “storm” possibly through the hepatic release of pro-inflammatory cytokines and by increased reactive oxygen production in COVID-19 patients (11–13).

There are still limited reports on how MAFLD and NAFLD influence clinical outcomes in patients with COVID-19, and there are no meta-analytical reports of the available evidence. This meta-analysis aimed to evaluate if MAFLD or NAFLD are associated with a more severe disease course of COVID-19.

## Methods and Materials

We report our systematic review and meta-analysis following the Preferred Reporting Items for Systematic Reviews and Meta-Analyses (PRISMA) 2009 Statement (Supplementary Table 1) (14). We registered the protocol of this study onto the International Prospective Register of Systematic Reviews (CRD42020210923) and adhered to it during the course, except for including mortality in our outcomes (see https://www.crd.york.ac.uk/prospero).

### Search and Selection

A systematic search was performed in five databases, namely Scopus, Cochrane Central Register of Controlled Trials (CENTRAL), Web of Science, Embase, and MEDLINE (via PubMed) without any search restrictions from inception to 15th Sept, 2020. The following search key was used: (NASH OR steatohepatitis OR “metabolic associated fatty liver disease” or “Non-alcoholic Fatty Liver Disease” OR “Nonalcoholic Fatty Liver Disease” OR MAFLD OR NAFLD) AND (“COVID 19” OR “Wuhan virus” OR “coronavirus” OR “2019 nCoV” OR “SARS-Cov-2”).

After the removal of duplicates with a reference manager software (EndNote X9, Clarivate Analytics, Philadelphia, PA, USA), papers for title, abstract, and full-text were screened by two independent authors separately according to a predetermined set of rules. In the case of any disagreement, a consensus was reached after discussion with a third author.

Eligible studies reported on (P) patients with confirmed SARS-CoV-2 infection and compared the outcomes of patients (I and C) with and without MAFLD or NAFLD to each other. The outcomes (O) were severe COVID-19, ICU admission, and in-hospital mortality. Studies with cohort or case-control design (>5 participants) were considered eligible. The severity of COVID-19 was classified according to the guidelines on the Diagnosis and Treatment of COVID-19 issued by the National Health Commission of China (Supplementary Table 2) (15). When there were multiple publications using data with overlapping study populations, we included the one with greater sample size.

### Data Extraction

Two independent review authors performed data extraction from eligible studies into a standardized data collection form. A third independent author resolved disagreements.

The following information was extracted from each study: first author, year of publication, digital object identifier, study design, study period, the number of centers, study site (country), demographic characteristics of the study population, the number of patients, the number of participants with and without MAFLD or NAFLD separately, the number of patients with event (severe COVID-19, ICU admission, mortality) with and without MAFLD or NAFLD separately, and, if available, odds ratios for COVID-19 severity, ICU admission, and mortality regarding MAFLD or NAFLD, and parameters included in multivariate adjustments.

### Statistical Analysis

All calculations were performed by Stata 15 data analysis and statistical software (Stata Corp LLC, College Station, TX, USA). All outcomes were handled as dichotomous variables, and odds ratios (ORs) with 95% confidence intervals (95% CIs) were calculated (reference groups: patients without NAFLD or MAFLD). Random effects model was used to calculate the pooled estimates using the DerSimonian-Laird method (16). A *p*-value of < 0.05 was considered statistically significant. Forest plots were used to present the results of the meta-analyses.

Heterogeneity was tested with *I*^2^ and χ^2^ tests. As suggested by the Cochrane Handbook (17), *I*^2^ values were interpreted as “might not be important” (0–40%), “moderate” (30–60%), “substantial” (50–90%), and “considerable” (75–100%) heterogeneity, with a *p* < 0.1 considered significant (18).

We were unable to assess the presence of publication bias because of the low number of studies included in each analysis.

### Assessment of Risk of Bias

Two independent review authors carried out the assessment. Discrepancies were resolved by third-party arbitration. We used the modified version of the Quality in Prognostic Studies (QUIPS) tool (19) as per the recommendations of the Cochrane Prognosis Methods Group (20). Methodological details of the assessment are summarized in Supplementary Appendix 1.

## Results

### Search and Selection

**Figure 1:**
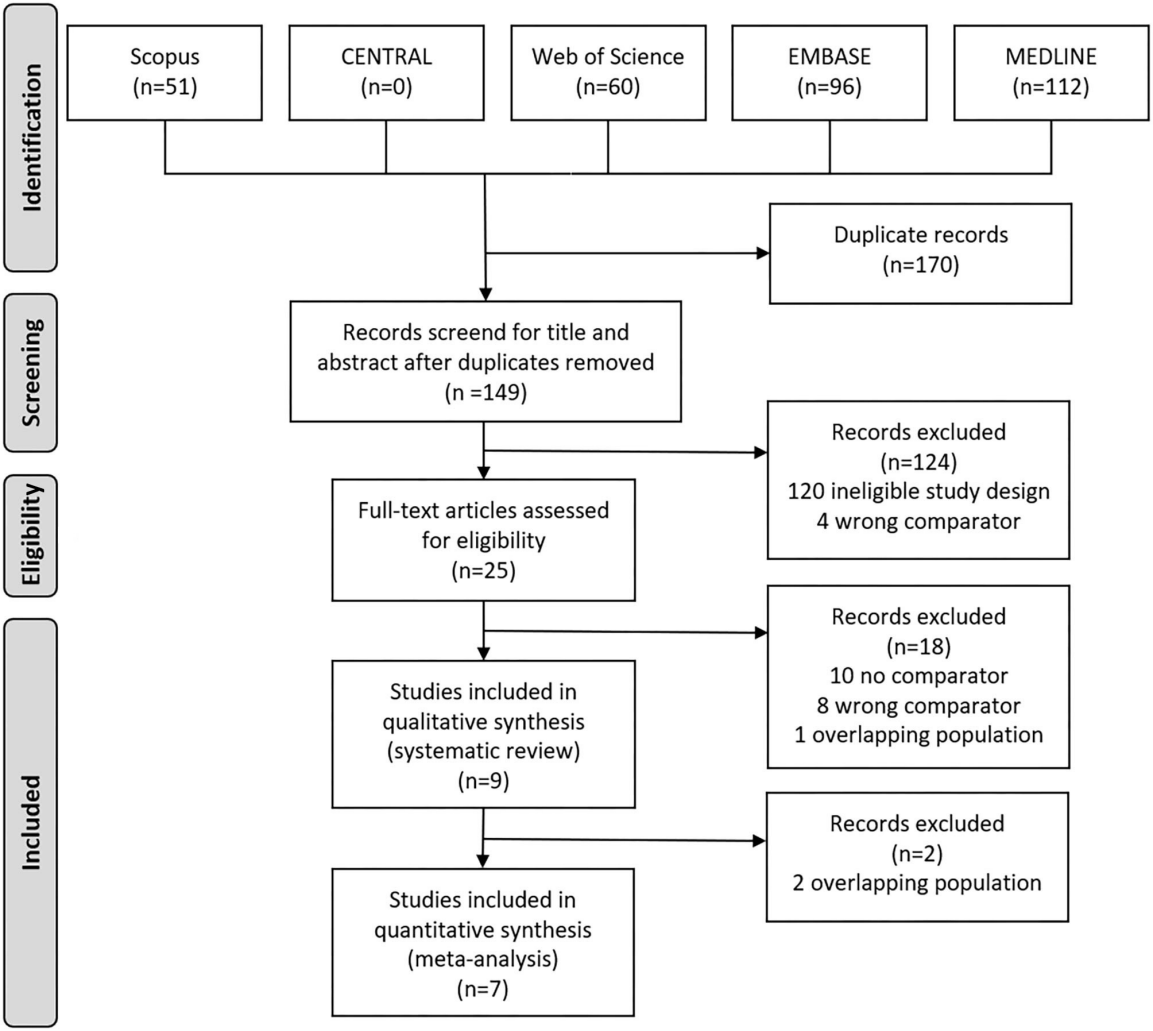
Preferred Reporting in Systematic Reviews and Meta-analyses (PRISMA) flowchart showing the selection process.

The selection process is detailed in Figure 1. We identified 319 records in five databases for evaluation. After the removal of duplicates and careful selection, 25 articles were eligible for full-text assessment. Altogether, 10 papers were eligible for qualitative and quantitative synthesis (9, 10, 21–27), however, we excluded one due to overlapping study population (12).

### Characteristics of the studies included

The main characteristics of the studies are summarized in Table 1. Two articles recruited subjects from the USA, one from Israel, and another six from China. Except for two prospective study, all were retrospective cohort studies. MAFLD was defined in all studies based on the consensus by Eslam et al. (8); NAFLD was defined by the presence of hepatic steatosis on imaging. The proportion of patients with MAFLD and NAFLD ranged from 28 to 50%, and from 6 to 38%, respectively, across studies. Eligibility criteria of the studies included are presented in Supplementary Table 3.

**Table 1:** Basic characteristics of included studies in the systematic review and meta-analysis.

**Author**	**Country**	**Total N^**0**^ of patients**	**Female%**	**Age (year)^†^**	**N^**0**^ of patients with MAFLD or NAFLD (% of total)**	**Outcome(s)**	
						**Definition**	**Event N^**0**^ (% of total)**
**NAFLD vs. no-NAFLD comparison**							
(9)	USA	6,802	44	46	373 (5.5)	Severe COVID-19/ ICU admission	930 (13.67)
							428 (6.3)
(23)	USA	351	45	63.4	57 (16)	ICU admission/ In-hospital mortality	132 (37.6)
							55 (15.67)
(22)	China	280	48	43	86 (31)	Severe COVID-19/ ICU admission	28 (10)
							18 (6.43)
(24)	China	202	44	44.5	76 (38)	Severe COVID-19	39 (19.31)
**MAFLD vs. no-MAFLD comparison**							
(27)	China	130	37	46	65 (50)	ICU admission	3 (2.31)
(25)	Israel	71	73	51	22 (31)	Severe COVID-19	13 (18.31)
(26)	China	310	52	47	94 (30)	Severe COVID-19	50 (16.13)
(21)^‡^	China	327	ND	ND	93 (28)	Severe COVID-19	59 (18)
(10)^‡^	China	110	26	42	55 (50)	ICU admission	3 (2.73)

† *mean or median*,

‡ *prospective study*.

*COVID-19, coronavirus disease 2019; ICU, intensive care unit; NAFLD, non-alcoholic fatty liver disease; ND, not defined; MAFLD, metabolic associated fatty liver disease*.

### Quantitative Syntheses

In our meta-analysis, we included a total of six studies with 7,284 patients evaluating the severity of COVID-19, the proportion of severe COVID-19 ranged from 10 to 19%. Three articles with 7,433 patients reported on the need for ICU admission, the proportion of ICU admission ranged from 6 to 38%.

**Figure 2:**
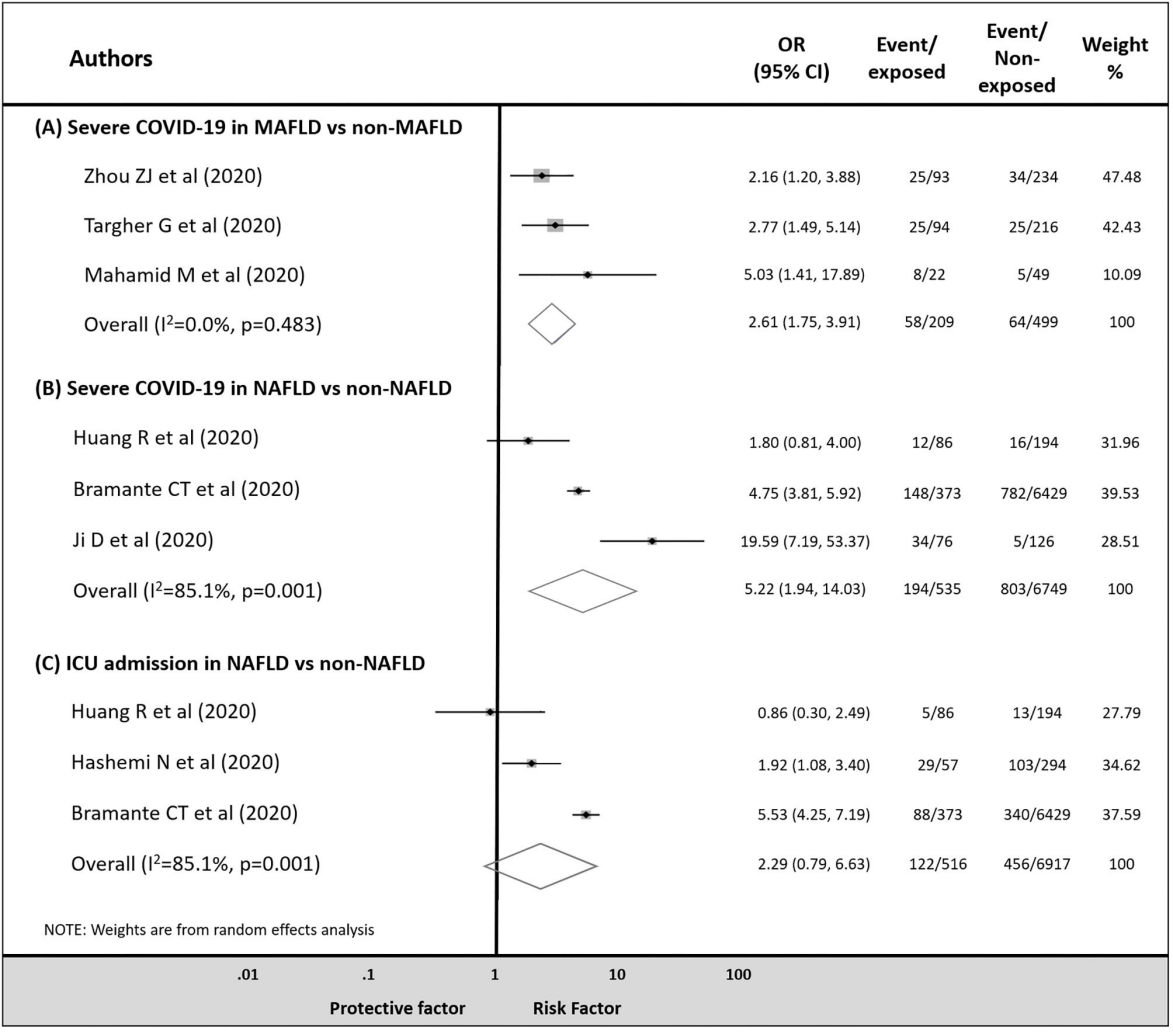
Odds ratio for COVID-19 severity in patients **(A)** with MAFLD vs. non-MAFLD, **(B)** with NAFLD vs. non-NAFLD, and odds ratio for ICU admission in patients **(C)** with NAFLD vs. non-NAFLD.

MAFLD was associated with an increased risk of severe COVID-19 compared to the non-MAFLD group [28 vs. 13%, respectively; OR = 2.61, CI: 1.75–3.91 in a homogenous dataset (*I*^2^ = 0.0% with *p* = 0.483)] (Figure 2A). Similarly, in the NAFLD vs. non-NAFLD comparison, NAFLD proved to be a risk factor as well [36 vs. 12%, respectively; OR = 5.22, CI: 1.94–14.03 in a heterogenous dataset (*I*^2^ = 85.1% with *p* = 0.001)] (Figure 2B).

Although patients with NAFLD were more likely to be admitted to ICU compared to those without NAFLD, the difference did not reach the level of statistical significance [24 vs. 7%, respectively; OR = 2.29, CI: 0.79–6.63 in a heterogenous dataset (*I*^2^ = 85.1% with *p* = 0.001)] (Figure 2C).

### Qualitative Syntheses

We were not able to make a meta-analytical analysis for the MAFLD vs. non-MAFLD comparison on the rate of ICU admission, however, two studies (10, 27) reported on ICU admission. Gao et al. (27) in non-diabetic MAFLD patients found an increased risk of intensive care requirement in those with critical illness compared to non-MAFLD patients (*p* = 0.003, 4.6 vs. 0.0%, respectively). Zhou et al. (10), in a matched cohort of MAFLD and non-MAFLD patients, found a significantly increased risk of the composite outcome of severe and critical COVID-19 in MAFLD patients compared to the non-MAFLD group (OR = 3.65, CI: 1.31–10.16).

Regarding in-hospital mortality, Hashemi et al. (23) found similar rates in COVID-19 patients with NAFLD compared to those without NAFLD (*p* = 0.54, 16.4 vs. 13.2%).

A summary of multivariate logistic regression analyses from each study included can be found in Supplementary Table 4. Most of the studies adjusted for age, sex, and underlying conditions in multivariate analysis. In the study of Ji et al. (24), NAFLD was associated with COVID-19 progression (adjusted OR = 6.4, CI: 1.5–31.2). Bramante et al. (9) found an increased odds of hospital admission in COVID-19 patients with NAFLD (adjusted OR = 2.04, CI: 1.55–2.69). Based on two studies, ICU admission (adjusted OR = 1.70, CI: 1.20–2.40; adjusted OR = 2.3, CI: 1.27–4.17, respectively) and need for mechanical ventilation (adjusted OR = 1.98, CI: 1.28–3.06; adjusted OR = 2.15, CI: 1.18–3.91, respectively) were also increased with NAFLD (9, 23). Finally, NAFLD was not found to increase in-hospital mortality in COVID-19 (adjusted OR = 0.99, CI: 0.54–1.77) (9).

On the other hand in COVID-19 patients with MAFLD, Mahamid et al. (25) found that MAFLD was associated with severe COVID-19 in both sexes (adjusted OR = 3.29, CI: 3.28–3.58 for men, adjusted OR = 3.25, CI: 3.09–3.47 for women), independently of MS. In the study of Zhou et al. (21), an association between the presence of MAFLD and COVID-19 severity was observed in patients younger than 60 years (adjusted OR = 2.67, CI: 1.13–6.34), but not in those above 60 years (adjusted OR = 0.61, CI: 0.18–2.03). In non-diabetic patients, Gao et al. (27) found an increased risk of severe COVID-19 only in MAFLD patients with both obesity and metabolic dysregulation (adjusted OR = 5.25, CI: 1.23–22.33), but the difference was non-significant if only one of the criteria was present (OR = 2.60, CI: 0.47–14.42).

### Risk of Bias Assessment

Among the included studies, three were of moderate overall risk of bias. All the other studies were rated to carry high overall risk of bias. The summary of risk of bias assessment is shown in Supplementary Figures 1–5.

## Discussion

In our meta-analysis, we aimed to analyse the association between MAFLD or NAFLD and COVID-19 outcomes. Based on our results, we identified that MAFLD is associated with 2.6 times higher risk of severe COVID-19 compared to the non-MAFLD group. In the NAFLD vs. non-NAFLD comparison, we found a five-times increased risk of severe COVID-19. The rate of the ICU admission was higher in NAFLD patients compared to those without NAFLD; however, the difference was statistically non-significant. Finally, we did not find any difference regarding in-hospital mortality in COVID-19 patients with MAFLD or NAFLD in qualitative synthesis.

Previous reviews have assessed the effect of MAFLD or NAFLD in COVID-19 patients, however, to our knowledge, this is the first systematic review and meta-analysis in this topic (6, 11, 28).

Six of the included articles reported on covariate adjusted results (9, 21, 23–25, 27), most of them supporting our conclusion on the impact of MAFLD and NAFLD in COVID-19. We could not perform a meta-analytical evaluation of these results, as there were different outcomes assessed and covariates adjusted for. Based on these results, MAFLD and NAFLD are associated with a higher risk of severe COVID-19 and ICU admission both in uni- and multi-variate analyses.

Previously several comorbidities such as hypertension, diabetes, extreme obesity, and cardiovascular disease were reported to be associated with worse prognosis in COVID-19 patients (3, 5). Several meta-analyses reported on the role of CLD in COVID-19 (29, 30). Based on our previous paper (31), pre-existing liver diseases and on-admission liver-related laboratory results predicted a more severe outcome in SARS-CoV-2 infection. However, none of the articles performed sub-group analysis based on the underlying liver condition.

The association between MAFLD or NAFLD and COVID-19 severity is certainly multifactorial. MS and elements of it have been already linked to untoward outcomes in COVID-19 (32). In type 2 diabetes, the second most common comorbidity in COVID-19, the poor prognosis is likely the consequence of the whole clinical picture: poor glucose control, advanced age, and diabetes-associated comorbidities (33). Obesity is associated with chronic inflammation compromising the immune response resulting in an increased risk of more severe infections (34, 35), on the other hand, obesity is also a significant risk factor for ICU admission and invasive mechanical ventilation (5). In patients with diabetes, hyperinflammatory response, microvascular endothelial dysfunction, and microthrombi formation may contribute to the poorer outcomes in COVID-19 (6).

Similarly, based on previous reports (26), in patients with MAFLD, a pro-inflammatory state could exacerbate the SARS-CoV-2 induced cytokine storm. Ji et al. (24) found in a retrospective study that COVID-19 patients with MAFLD had a poorer prognosis, two-fold higher prevalence of severe disease course, and also higher viral shedding time, and more liver failure during hospitalization.

In the included studies several differences between study populations were highlighted. Increased liver fat content was associated with a higher risk of symptomatic COVID-19 in univariate analysis (OR = 1.85, 95% OR: 1.05–3.25) (36). Moreover, the authors found that obesity and concomitant >10% liver fat content exposed an increased risk of severe COVID-19 (OR = 2.96, 95% CI: 1.12–7.78); those obese patients with normal liver fat content (<5%) showed no elevation of risk (OR = 0.36, 95% CI: 0.1–1.26). The importance of the liver fat content has been pointed out in the study by Bramante et al. (9) as well.

On the other hand, the presence of fibrosis in MAFLD patients is another risk factor for severity of COVID-19, independently of metabolic comorbidities. Based on Targher et al. (12), the severity of COVID-19 significantly increased with the extent of liver fibrosis; those with a FIB-4 score higher than 2.67 had the highest risk of developing severe COVID-19 (OR = 5.73, 95% CI: 1.84–17.9). After adjustment for sex, obesity, and diabetes, this considerable association persisted (adjusted OR = 2.91, 95% CI: 1.20–7.06).

The same authors demonstrated that the presence of MAFLD together with a neutrophil-to-lymphocyte ratio (NLR) higher than 2.8 is associated with a higher risk of severe COVID-19 compared to patients without MAFLD and with normal NLR (26). NLR was previously highlighted to be a useful, widely available prognostic factor in the early phase of SARS-CoV-2 infection (37).

Another interesting point was reported by Zhou et al. (21). In COVID-19 patients with MAFLD under 60 years, a more than 4-fold risk of severe COVID-19 was observed compared to those without MAFLD (OR = 3.97, 95% CI: 1.89–8.35); after adjusting for covariates (adjusted OR = 2.67, 95% CI: 1.13–6.34) the risk remained significantly higher. In contrast, in multivariate analysis in elderly patients, MAFLD was not associated with severity of COVID-19. These results need to be supported by further cohort analysis.

None of the studies reported on long-term outcomes in COVID-19.

### Strengths and Limitations

Considering the strengths of our meta-analysis, a rigorous methodology was followed, and we did not deviate from the pre-study protocol, except for including mortality in our investigated outcomes. Several limitations must be considered when interpreting our results. First of all, we could not analyse in-hospital mortality in our meta-analysis. Secondly, our study involved data from only nine articles. It must be noted that, we detected significant differences despite the limited study populations, however, with considerable statistical heterogeneity in some of our results. Most of the studies included a low number of patients. The number of studies prevented us from analyzing publication bias (<10 articles). Most of the articles were published from Asian countries; therefore, it is difficult to generalize these results. Also the rate of MAFLD and NAFLD in the study populations differed from the rate reported in the general population. The definition of MAFLD was homogenous, however, NAFLD was diagnosed using different methods across studies. Finally, data came mostly from retrospective studies, with most of them carrying high risk of bias.

## Conclusion

### Implication for Practice

In conclusion, the presence of MAFLD or NAFLD is associated with a more severe COVID-19. The presence of further metabolic dysfunction may have additional negative impact on the course of COVID-19. Based on this, health-care providers should follow MAFLD patients cautiously and preventive measures should be taken in these high-risk populations. Therefore, weight loss and regular physical activity should be encouraged in MAFLD patients.

### Implication for Research

The underlying mechanisms behind our results are still poorly understood. Further research is needed to understand the effect of the pro-inflammatory state associated with MAFLD on the cytokine storm caused by SARS-CoV-2 infection. The severity of COVID-19 should be further stratified based on the severity of MAFLD to explore further high-risk patient groups. Further research is needed to support our results as well as other outcomes, such as mortality, should be analyzed.

## Data Availability Statement

The original contributions generated for the study are included in the article/Supplementary Material, further inquiries can be directed to the corresponding author/s.

## Author Contributions

GP and PJH designed the research and the study concept. ZS and SV performed the data extraction. NF analyzed and interpreted the data. FD and KO performed the quality and risk assessment, PJH, BE, SV, SK, PH, and GP wrote the article. BE, PH, and GP conducted a critical revision of the manuscript for important intellectual content. All of the co-authors granted final approval of the version of the article to be published.

## Conflict of Interest

The authors declare that the research was conducted in the absence of any commercial or financial relationships that could be construed as a potential conflict of interest.
