# Supplementary material for: Metabolic Associated Fatty Liver Disease Is Associated With an Increased Risk of Severe COVID-19: A Systematic Review With Meta-Analysis
Source: Front Med (Lausanne). 2021 Mar 12;8:626425. doi: 10.3389/fmed.2021.626425 (PMC7994270; doi:10.3389/fmed.2021.626425)
Supplement: Supplementary file 1 [file Table_1.DOCX]

Supplementary Material

Metabolic associated fatty liver disease is associated with an increased risk of severe COVID-19: A systematic review with meta-analysis

Authors and affiliation

**Péter Jenő Hegyi^1,^** ^†^**, Szilárd Váncsa^1,2,^** ^†^**, Klementina Ocskay^1,2^, Fanni Dembrovszky^1,2^, Szabolcs Kiss^1,2,3^, Nelli Farkas^1,4^, Bálint Erőss^1^, Zsolt Szakács^1,2^, Péter Hegyi^1,2^, Gabriella Pár^1,5^**

^1^ Institute for Translational Medicine, Medical School, University of Pécs, Pécs, Hungary

^2^ Szentágothai Research Center, University of Pécs, Pécs, Hungary

^3^ Doctoral School of Clinical Medicine, University of Szeged, Szeged, Hungary

^4^ Institute of Bioanalysis, Medical School, University of Pécs, Pécs, Hungary

^5^ Division of Gastroenterology, First Department of Medicine, Medical School, University of Pécs, Pécs, Hungary

^†^authors contributed equally to the work

# Table of contents

**Supplementary Table 1.** PRISMA checklist

**Supplementary Table 2.** Classification of COVID-19 severity

**Supplementary Table 3.** Eligibility criteria in each included article in the systematic review and meta-analysis

**Supplementary Table 4.** Multivariate analysis on the effect of MAFLD/NAFLD on COVID-19 outcomes

**Supplementary Appendix 1.** Risk of bias assessment using the QUIPS tool

**Supplementary Figure 1-3.** Risk of bias assessment using the QUIPS tool

**References**

# Supplementary Tables

Supplementary Table 1. PRISMA checklist(1)

| **Section/topic** | **#** | **Checklist item** | **Reported on page #** |
| --- | --- | --- | --- |
| **TITLE** | | |  |
| Title | 1 | Identify the report as a systematic review, meta-analysis, or both. | 1 |
| **ABSTRACT** | | |  |
| Structured summary | 2 | Provide a structured summary including, as applicable: background; objectives; data sources; study eligibility criteria, participants, and interventions; study appraisal and synthesis methods; results; limitations; conclusions and implications of key findings; systematic review registration number. | 1-2 |
| **INTRODUCTION** | | |  |
| Rationale | 3 | Describe the rationale for the review in the context of what is already known. | 2 |
| Objectives | 4 | Provide an explicit statement of questions being addressed with reference to participants, interventions, comparisons, outcomes, and study design (PICOS). | 2 |
| **METHODS** | | |  |
| Protocol and registration | 5 | Indicate if a review protocol exists, if and where it can be accessed (e.g., Web address), and, if available, provide registration information including registration number. | 2 |
| Eligibility criteria | 6 | Specify study characteristics (e.g., PICOS, length of follow-up) and report characteristics (e.g., years considered, language, publication status) used as criteria for eligibility, giving rationale. | 3 |
| Information sources | 7 | Describe all information sources (e.g., databases with dates of coverage, contact with study authors to identify additional studies) in the search and date last searched. | 3 |
| Search | 8 | Present full electronic search strategy for at least one database, including any limits used, such that it could be repeated. | 3 |
| Study selection | 9 | State the process for selecting studies (i.e., screening, eligibility, included in systematic review, and, if applicable, included in the meta-analysis). | 3 |
| Data collection process | 10 | Describe method of data extraction from reports (e.g., piloted forms, independently, in duplicate) and any processes for obtaining and confirming data from investigators. | 3 |
| Data items | 11 | List and define all variables for which data were sought (e.g., PICOS, funding sources) and any assumptions and simplifications made. | 3 |
| Risk of bias in individual studies | 12 | Describe methods used for assessing risk of bias of individual studies (including specification of whether this was done at the study or outcome level), and how this information is to be used in any data synthesis. | 4 |
| Summary measures | 13 | State the principal summary measures (e.g., risk ratio, difference in means). | 3 |
| Synthesis of results | 14 | Describe the methods of handling data and combining results of studies, if done, including measures of consistency (e.g., I^2^) for each meta-analysis. | 3 |
| Risk of bias across studies | 15 | Specify any assessment of risk of bias that may affect the cumulative evidence (e.g., publication bias, selective reporting within studies). | 4 |
| Additional analyses | 16 | Describe methods of additional analyses (e.g., sensitivity or subgroup analyses, meta-regression), if done, indicating which were pre-specified. | - |
| **RESULTS** | | |  |
| Study selection | 17 | Give numbers of studies screened, assessed for eligibility, and included in the review, with reasons for exclusions at each stage, ideally with a flow diagram. | 4 |
| Study characteristics | 18 | For each study, present characteristics for which data were extracted (e.g., study size, PICOS, follow-up period) and provide the citations. | 4, Table 1 |
| Risk of bias within studies | 19 | Present data on risk of bias of each study and, if available, any outcome level assessment (see item 12). | Suppl. Figure 1-5 |
| Results of individual studies | 20 | For all outcomes considered (benefits or harms), present, for each study: (a) simple summary data for each intervention group (b) effect estimates and confidence intervals, ideally with a forest plot. | Table 1 |
| Synthesis of results | 21 | Present results of each meta-analysis done, including confidence intervals and measures of consistency. | 4-5 |
| Risk of bias across studies | 22 | Present results of any assessment of risk of bias across studies (see Item 15). | 5 |
| Additional analysis | 23 | Give results of additional analyses, if done (e.g., sensitivity or subgroup analyses, meta-regression [see Item 16]). | - |
| **DISCUSSION** | | |  |
| Summary of evidence | 24 | Summarize the main findings including the strength of evidence for each main outcome; consider their relevance to key groups (e.g., healthcare providers, users, and policy makers). | 5-7 |
| Limitations | 25 | Discuss limitations at study and outcome level (e.g., risk of bias), and at review-level (e.g., incomplete retrieval of identified research, reporting bias). | 7 |
| Conclusions | 26 | Provide a general interpretation of the results in the context of other evidence, and implications for future research. | 7 |
| **FUNDING** | | |  |
| Funding | 27 | Describe sources of funding for the systematic review and other support (e.g., supply of data); role of funders for the systematic review. | 9 |

**Supplementary Table 2.** Classification of COVID-19 severity (2)

| **Severity** | **Definition** |
| --- | --- |
| Mild | The clinical symptoms are mild with no abnormal radiological findings. |
| Moderate | Fever, cough and other symptoms are presented with pneumonia on chest computed tomography. |
| Severe | One of the following conditions is met: (1) Respiratory distress, respiratory rate ≥ 30 per min; (2) Oxygen saturation on room air at rest ≤ 93%; (3) Partial pressure of oxygen in arterial blood / fraction of inspired oxygen ≤ 300 mmHg. |
| Critical | One of the following conditions has to be met: (1) Respiratory failure occurs and mechanical ventilation is required; (2) Shock occurs; (3) Patients with other organ dysfunction needing intensive care unit monitoring. |

**Supplementary Table 3.** Eligibility criteria in each included article in the systematic review and meta-analysis

| **Author** | **Outcome(s)** | **Outcome(s) definition** | **NAFLD/MAFLD definition** | **COVID-19 diagnosis** |
| --- | --- | --- | --- | --- |
| Bramante CT et al (3) | Severe COVID-19/ ICU admission | Not defined | NAFLD: ICD codes for NAFLD or NASH or a BMI >= 30kg/m2 and an elevated alanine aminotransferase (ALT) on 3 separate dates. | PCR-confirmed |
| Gao F et al (4) | ICU admission | COVID-19 severity of illness was assessed during hospitalization and classified into four clinical subtypes (i.e. mild, moderate, severe, and critically ill) on the basis of management guidelines. | MAFLD: Criteria by Eslam et al. 2020 (5). Authors excluded patients with confirmed diabetes. | PCR-confirmed |
| Hashemi N et al (6) | ICU admission/ in-hospital mortality | Not defined | NAFLD: The definition of NAFLD per AASLD (7) | PCR-confirmed |
| Huang R et al (8) | Severe COVID-19/ ICU admission | Not defined | NAFLD: Using the published hepatic steatosis index (HSI) in the absence of other causes of CLD. This index was calculated by using the following equation: HSI = 8 × ( ALT/AST ratio) + body mass index (BMI) (+2, if a female patient; +2, if diabetic). Serum ALT and AST results of the first test after admission were used for the calculation of the HSI. A cutoff of 36 was used to define the presence of NAFLD. | PCR-confirmed |
| Ji D et a (9)l | Severe COVID-19 | In accordance with the practice guidelines issued by The Chinese National Health Commission.  Progression of illness was defined as development of at least one of the following: respiratory rate >30 breaths/min, resting oxygen saturation <93% and PaO2/FiO2 <300 mmHg or worsening of lung CT findings, during the hospitalization period. | NAFLD: hepatic steatosis index (HSI = 8 × [ALT/ AST] + BMI [+ 2 if type 2 diabetes yes, + 2 if female]) >36 points and/or by abdominal ultrasound examination | PCR-confirmed |
| Mahamid M et al (10) | Severe COVID-19 | COVID-19 severity was assessed according to diagnosis and treatment protocol for novel Coronavirus pneumonia released by National Health Commission & State Administration of Traditional Chinese Medicine on 3 March 2020 (trial version 7)  Severe cases: respiratory distress (equal or more than 30 breaths per minute), oxygen saturation equal or below 93% at rest, arterial partial pressure of oxygen PaO2/fraction of inspired oxygen (FiO2) equal or less than 300 mmHg. | MAFLD: Criteria by Eslam et al. 2020 (5) | PCR-confirmed |
| Targher G et al (11) | Severe COVID-19 | The severity of COVID-19 was assessed during hospitalisation and classified as severe and non-severe based on the current management guideline | MAFLD: Criteria by Eslam et al. 2020 (5) | PCR-confirmed |
| Zhou YJ et al (12) | Severe COVID-19 | Details in article by Zhou YJ et al (13): COVID-19 severity was assessed during hospitalization and classified into four clinical subtypes (mild, moderate, severe and critical), based on the Chinese management guideline. We defined mild and moderate COVID-19 subtypes as ‘non-severe COVID-19’, and severe and critical subtypes as ‘severe COVID-19’. | MAFLD: Criteria by Eslam et al. 2020 (5) | PCR-confirmed |
| Zhou YJ et al (13) | ICU admission | COVID-19 severity was assessed during hospitalization and classified into four clinical subtypes (mild, moderate, severe and critical), based on the Chinese management guideline. We defined mild and moderate COVID-19 subtypes as ‘non-severe COVID-19’, and severe and critical subtypes as ‘severe COVID-19’. | MAFLD: Criteria by Eslam et al. 2020 (5) | PCR-confirmed |

For classification of COVID-19 see Supplementary Table 3.

ICU: intensive care unit, NAFLD: non-alcoholic fatty liver disease, NASH: non-alcoholic steatohepatitis, MAFLD: metabolic associated fatty liver disease, PCR: polymerase chain reaction

**Supplementary Table 4.** Multivariate analysis on the effect of MAFLD/NAFLD on COVID-19 outcomes

| **Author** | **Odds ratio** | **95% Confidence interval** | **Parameters included in multivariate analysis** |  |
| --- | --- | --- | --- | --- |
| **Severe COVID-19 in patients with NAFLD** | | | | |
| Bramante CT et al (3) | 2.04 | 1.55-2.69 | Age, sex, obesity, ethnicity, NAFLD/NASH, alcohol use disorder, Elixhauser comorbidity index, and home use of amiodarone, methotrexate, oral steroids, or calcium channel blockers (CCB) |  |
| Ji D et al (9) | 6.4 | 1.5-31.2 | Sex, age>60 years, higher BMI, underlying comorbidities and NAFLD |  |
| **ICU admission in COVID-19 in patients with NAFLD** | | | | |
| Bramante CT et al | 1.70 | 1.20-2.40 | Age, sex, obesity, ethnicity, NAFLD/NASH, alcohol abuse, Elixhauser comorbidity index, and home medications (amiodarone, methotrexate, oral steroids, or calcium channel blockers). |  |
| Hashemi N et al (6) | 2.30 | 1.27-4.17 | Age, gender, BMI, cardiac disease, hyperlipidemia, hypertension, diabetes, pulmoary diseases and NAFLD |  |
| **Need for mechanical ventilation or intubation in COVID-19 in patients with NAFLD** | | | | |
| Bramante CT et al | 1.98 | 1.28-3.06 | Age, sex, obesity, ethnicity, NAFLD/NASH, alcohol abuse, Elixhauser comorbidity index, and home medications (amiodarone, methotrexate, oral steroids, or calcium channel blockers). |  |
| Hashemi N et al (6) | 2.15 | 1.18-3.91 | Age, gender, BMI, cardiac disease, hyperlipidemia, hypertension, diabetes, pulmoary diseases and NAFLD |  |
| **In-hospital mortality in patients with NAFLD** | | | | |
| Bramante CT et al | 0.99 | 0.54-1.77 | Age, sex, obesity, ethnicity, NAFLD/NASH, alcohol abuse, Elixhauser comorbidity index, and home medications (amiodarone, methotrexate, oral steroids, or calcium channel blockers). |  |
| **Severe COVID-19 in patients with MAFLD** | | | | |
| Mahamid M et al (10) | 3.29 | 3.28-3.58 | Male gender; MAFLD, obesity, hypertension, metabolic syndrome diabetes and smoking |  |
| Mahamid M et al (10) | 3.25 | 3.09-3.47 | Women gender; MAFLD, obesity, hypertension, metabolic syndrome diabetes and smoking |  |
| Zhou YJ et al (12) | 2.49 | 1.04-5.96 | Age<60, sex, smoking, obesity, diabetes mellitus, hypertension and MAFLD |  |
| **Severe and critical COVID-19 in patients with MAFLD** | | | | |
| Gao F et el (4) | 4.07 | 1.10-15.09 | Age, sex, smoking status, obesity, hypertension, dyslipidemia and MAFLD |  |

BMI: body mass index, NAFLD: non-alcoholic fatty liver disease, NASH: non-alcoholic steatohepatitis, MAFLD: metabolic associated fatty liver disease

# Supplementary Appendix 1. Modified QUIPS tool methods (14)

Results of the modified QUIPS score. Low risk of bias was provided in the case of statistical analysis reporting domain, because all included articles reported raw data for our analysis. Study attrition was assessed in the case of prospective cohort studies We waived the risk assessment of study confounding. Study participation, prognostic factor and outcome measurement were assessed in every included article.

Study participation measurement: Low risk of bias was provided if study participant selection process and basic characteristics was described. Unclear risk of bias was attribute if these details were not reported.

Prognostic factor measurement: Low risk of bias was given if a clear definition of the prognostic factor was provided. In the case of unclear risk of bias no information about the definition of the prognostic factor was available. Studies, which described a definition not according to the international definitions of prognostic factors were defined as articles of high risk.

Outcome measurement: Low risk of bias was given if a clear definition, according to the accepted guidelines was provided. In the case of unclear risk of bias no information about the definition of the outcome was available. Studies, which described a definition not according to the accepted definitions of outcomes were defined as high risk carrying articles.

# Supplementary Figures


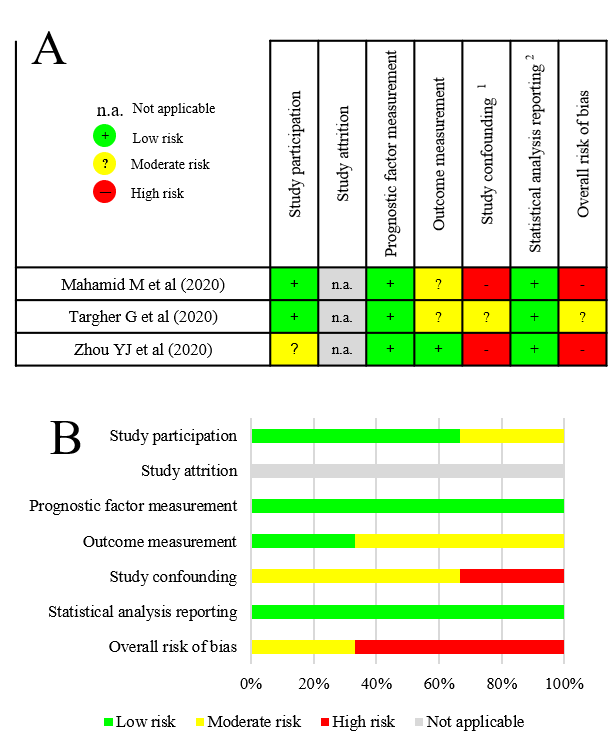
**Supplementary Figure 1.** Risk of bias assessment on study level [A] and across studies [B] for MAFLD patients with severe COVID-19.

Assessed confounding factors are age, hypertension, heart failure, pulmonary disease, smoking and alcohol consumption. Differences in obesity and type 2 diabetes were not considered, as they are closely linked with MAFLD. Only studies reporting the number of patients still hospitalized were judged to carry low risk of bias for outcome measurement.


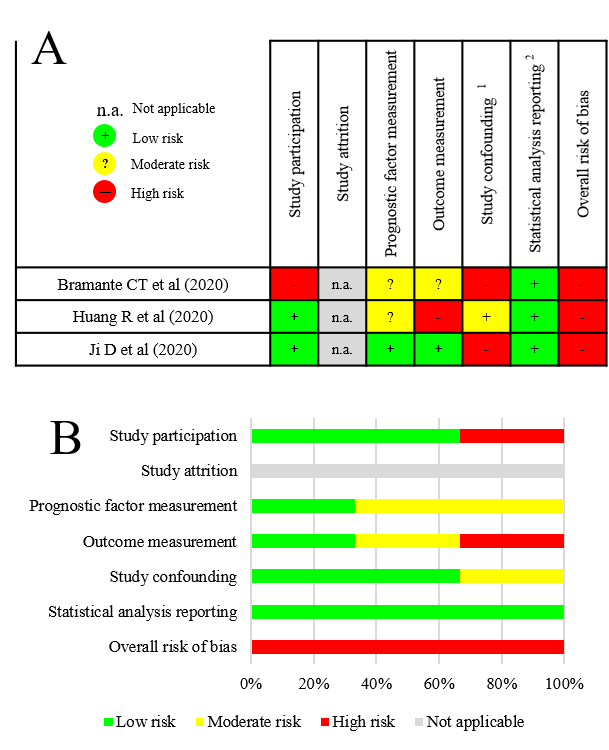
**Supplementary Figure 2.** Risk of bias assessment on study level [A] and across studies [B] for NAFLD patients with severe COVID-19.

Assessed confounding factors are age, hypertension, heart failure, pulmonary disease, smoking and alcohol consumption. Differences in obesity and type 2 diabetes were not considered, as they are closely linked with NAFLD. Only studies reporting the number of patients still hospitalized were judged to carry low risk of bias for outcome measurement.


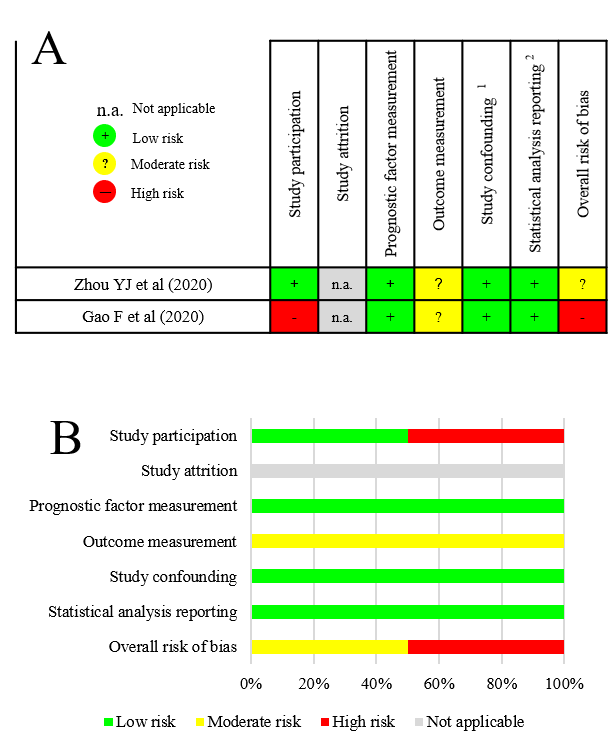
**Supplementary Figure 3.** Risk of bias assessment on study level [A] and across studies [B] in MAFLD patients with COVID-19 requiring intensive care

Assessed confounding factors are age, hypertension, heart failure, pulmonary disease, smoking and alcohol consumption. Differences in obesity and type 2 diabetes were not considered, as they are closely linked with MAFLD. Only studies reporting the number of patients still hospitalized were judged to carry low risk of bias for outcome measurement.


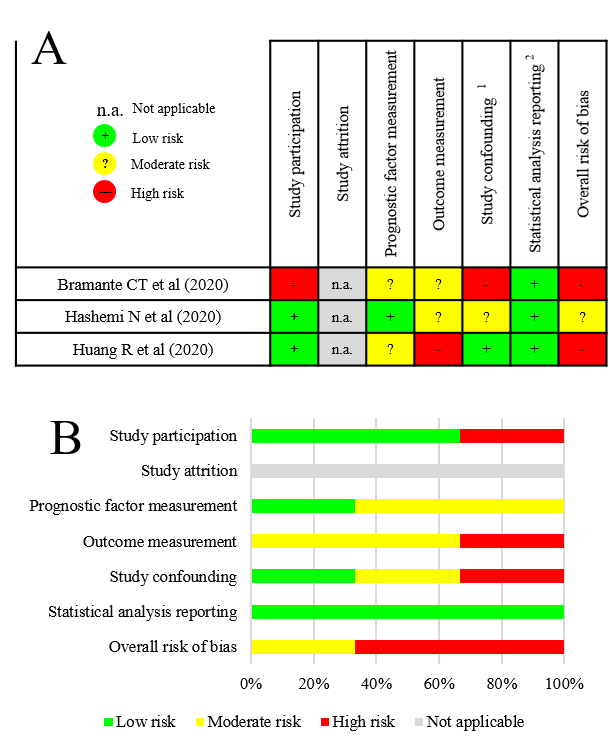
**Supplementary Figure 4.** Risk of bias assessment on study level [A] and across studies [B] NAFLD patients with COVID-19 requiring intensive care.

Assessed confounding factors are age, hypertension, heart failure, pulmonary disease, smoking and alcohol consumption. Differences in obesity and type 2 diabetes were not considered, as they are closely linked with NAFLD. Only studies reporting the number of patients still hospitalized were judged to carry low risk of bias for outcome measurement.


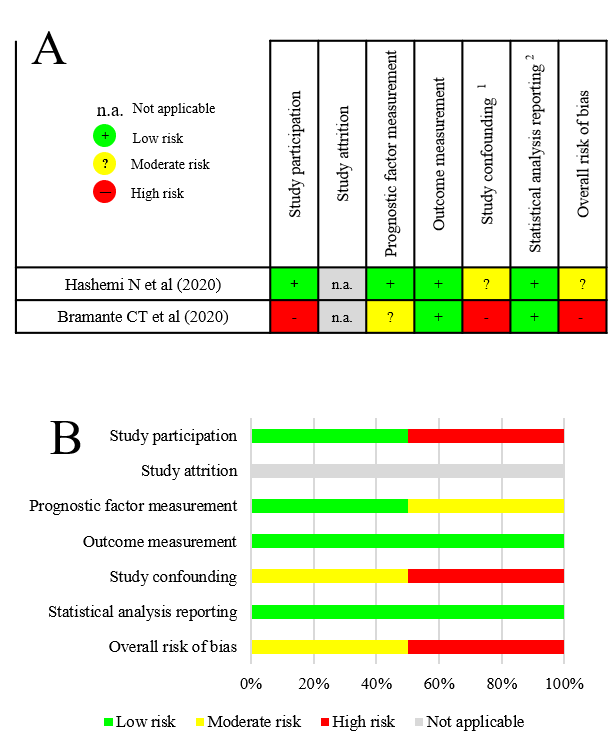
**Supplementary Figure 5.** Risk of bias assessment on study level [A] and across studies [B] in NAFLD patients with COVID-19 regarding in-hospital mortality.

Assessed confounding factors are age, hypertension, heart failure, pulmonary disease, smoking and alcohol consumption. Differences in obesity and type 2 diabetes were not considered, as they are closely linked with NAFLD. Only studies reporting the number of patients still hospitalized were judged to carry low risk of bias for outcome measurement.

# REFERENCES

1. Moher D, Liberati A, Tetzlaff J, Altman DG. Preferred reporting items for systematic reviews and meta-analyses: the PRISMA statement. *BMJ*. (2009) 339:b2535. doi: 10.1136/bmj.b2535

2. Zu ZY, Jiang MD, Xu PP, Chen W, Ni QQ, Lu GM, et al. Coronavirus Disease 2019 (COVID-19): A Perspective from China. *Radiology*. (2020) 296(2):E15-e25. doi: 10.1148/radiol.2020200490

3. Bramante C, Tignanelli CJ, Dutta N, Jones E, Tamariz L, Clark JM, et al. Non-alcoholic fatty liver disease (NAFLD) and risk of hospitalization for Covid-19. *medRxiv*. (2020). doi: 10.1101/2020.09.01.20185850

4. Gao F, Zheng KI, Wang X-B, Yan H-D, Sun Q-F, Pan K-H, et al. Metabolic associated fatty liver disease increases coronavirus disease 2019 disease severity in nondiabetic patients. *J Gastroenterol Hepatol*. (2020). doi: 10.1111/jgh.15112

5. Eslam M, Sanyal AJ, George J. MAFLD: A Consensus-Driven Proposed Nomenclature for Metabolic Associated Fatty Liver Disease. *Gastroenterology*. (2020) 158(7):1999-2014.e1. doi: 10.1053/j.gastro.2019.11.312

6. Hashemi N, Viveiros K, Redd WD, Zhou JC, McCarty TR, Bazarbashi AN, et al. Impact of chronic liver disease on outcomes of hospitalized patients with COVID-19: A multicentre United States experience. *Liver Int*. (2020) 40(10):2515-21. doi: 10.1111/liv.14583

7. Chalasani N, Younossi Z, Lavine JE, Charlton M, Cusi K, Rinella M, et al. The diagnosis and management of nonalcoholic fatty liver disease: Practice guidance from the American Association for the Study of Liver Diseases. *Hepatology*. (2018) 67(1):328-57. doi: 10.1002/hep.29367

8. Huang R, Zhu L, Wang J, Xue L, Liu L, Yan X, et al. Clinical Features of Patients With COVID-19 With Nonalcoholic Fatty Liver Disease. *Hepatol Commun*. (2020). doi: 10.1002/hep4.1592

9. Ji D, Qin E, Xu J, Zhang D, Cheng G, Wang Y, et al. Non-alcoholic fatty liver diseases in patients with COVID-19: retrospective study. *J Hepatol*. (2020) 73(2):451-3. doi: 10.1016/j.jhep.2020.03.044

10. Mahamid M, Nseir W, Khoury T, Mahamid B, Nubania A, Sub-Laban K, et al. Nonalcoholic fatty liver disease is associated with COVID-19 severity independently of metabolic syndrome: a retrospective case-control study. *Eur J Gastroenterol Hepatol*. (2020). doi: 10.1097/meg.0000000000001902

11. Targher G, Mantovani A, Byrne CD, Wang XB, Yan HD, Sun QF, et al. Detrimental effects of metabolic dysfunction-associated fatty liver disease and increased neutrophil-to-lymphocyte ratio on severity of COVID-19. *Diabetes Metab Syndr*. (2020). doi: 10.1016/j.diabet.2020.06.001

12. Zhou Y-J, Zheng KI, Wang X-B, Yan H-D, Sun Q-F, Pan K-H, et al. Younger patients with MAFLD are at increased risk of severe COVID-19 illness: A multicenter preliminary analysis. *J Hepatol*. (2020) 73(3):719-21. doi: 10.1016/j.jhep.2020.04.027

13. Zhou Y-J, Zheng KI, Wang X-B, Sun Q-F, Pan K-H, Wang T-Y, et al. Metabolic-associated fatty liver disease is associated with severity of COVID-19. *Liver Int*. (2020) 40(9):2160-3. doi: 10.1111/liv.14575

14. Hayden JA, van der Windt DA, Cartwright JL, Côté P, Bombardier CJAoim. Assessing bias in studies of prognostic factors. *Ann Intern Med*. (2013) 158(4):280-6. doi: 10.7326/0003-4819-158-4-201302190-00009
